# Comparative study on organoleptic properties and volatile organic compounds in turmeric, turmeric essential oil, and by-products using E-nose, HS-GC-IMS, and HS-GC-MS

**DOI:** 10.1016/j.fochx.2024.102107

**Published:** 2024-12-28

**Authors:** Bing Yang, Wanjia Wang, Jianuo Zhang, Wei Gao, Lipeng Fan, Bimal Chitrakar, Yaxin Sang

**Affiliations:** a Collage of Food Science and Technology, Hebei Agricultural University, Baoding, China; b Chen Guang Biotechnology Group Co., Ltd., Handan, China

**Keywords:** *Curcuma longa*, Color, Sensory, VOC, Gas-chromatography

## Abstract

The properties, applications, and in vitro bioactivities of turmeric, turmeric essential oil (TEO), and turmeric essential oil by-products (TEO-BP) were evaluated using sensory analysis, gas chromatography–mass spectrometry (GC–MS), gas chromatography-ion mobility spectrometry (GC-IMS), and electronic nose techniques. A total of 62 and 66 volatile organic compounds (VOCs), primarily terpenoids and sesquiterpenoids, were identified by GC–MS and GC-IMS, respectively. Distillation temperature, particularly at 90 °C, significantly influenced the color and organoleptic properties of TEO, with variations in VOC profiles driving these differences. Molecular distillation at 90 °C was found to optimize the purification and concentration of key VOCs in TEO. All turmeric samples demonstrated robust antioxidant and α-glucosidase inhibitory activities, with TEO-90 exhibiting the highest bioactivity. These results underscore the potential applications of TEO and TEO-BP in food and nutraceutical industries, offering a sustainable strategy to reduce waste and enhance the efficient utilization of turmeric resources.

## Highlights

• Identified 20 key VOCs to differentiate turmeric samples using VIP analysis.
• Established 90 °C as optimal temperature for TEO extraction via distillation.
• Combined GC–MS, GC-IMS, and *E*-nose to analyze 77 VOCs in turmeric samples.
• Demonstrated TEO-BP enrichment in antioxidant and α-glucosidase activities.
• Showed VOCs' impact on turmeric color and sensory characteristics.

## Graphical abstract

**Figure:**
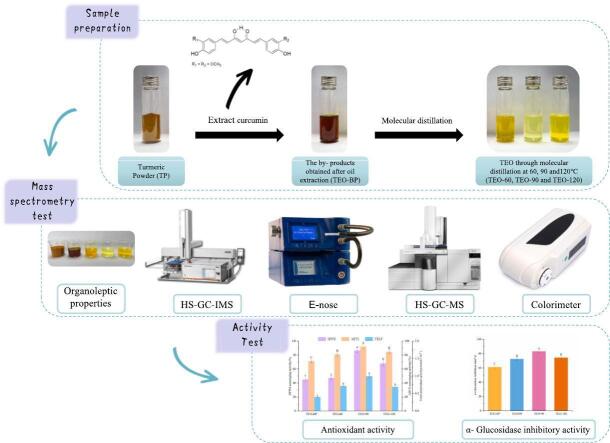

## 1 Introduction

Turmeric (*Curcuma longa*) is a perennial medicinal herb in the *Curcuma* genus and the family *Zingiberaceae* (Yue et al., 2015). Native to South and Southeast Asia, turmeric is now widely cultivated in countries including India, Bangladesh, China, Thailand, Cambodia, Malaysia, the Philippines, and Indonesia. Turmeric primarily contains curcuminoids, essential oils, and resin, making it a globally significant commodity (Nair, 2013). Renowned for its distinctive flavor, turmeric is extensively used as a spice, natural dye, preservative, and household ingredient (Megumi et al., 2017). India dominates global production, export, and consumption of turmeric, which is also utilized across diverse sectors such as medicine, food, and health care (Pushpa et al., 2022; Soleimani et al., 2018).

Curcuminoids, the principal bioactive compounds in turmeric, are orange-yellow crystalline phenolic substances with a bitter taste. Although insoluble in water, they dissolve readily in alcohol (Wang et al., 2022). Recent advancements in pH-responsive freshness indices employing natural food colorants have identified curcuminoids as potential acid-base indicators for monitoring the freshness of perishable foods (Priyadarshi et al., 2021). Industrially, curcuminoids are extracted from turmeric oleoresin, a concentrated turmeric extract. The residue left after curcuminoid extraction is termed Curcumin Removed Turmeric Oleoresin (CRTO), which contains volatile and non-volatile oils, resin, and residual curcuminoids (Nagarajan et al., 2010). The essential oils in CRTO are rich in bioactive compounds (Jayaprakasha et al., 2014; Kumar & Subramanian, 2020). According to literature reports, the distillation temperature range of plant essential oil using molecular distillation process is 50–150 °C (Guo et al., 2022; Reddy Sagili et al., 2023; Zhang et al., 2024).Turmeric essential oil (TEO) primarily comprises turmerones, turmericenes, and sesquiterpenes (Kuttan et al., 2011). TEO has been shown to promote wound healing, alleviate type II diabetes symptoms, lower blood pressure, and reduce cholesterol levels (Jy et al., 2021). Additionally, emerging evidence suggests turmeric may support brain repair and recovery in neurodegenerative disorders. These findings underscore turmeric's extensive health benefits, enhancing both physical well-being and daily life. While curcumin is widely recognized as turmeric's key bioactive compound, other components with diverse biological activities remain underexplored, leading to limited utilization of turmeric's full potential (Aggarwal et al., 2013). For instance, a study analyzing turmeric powder (TP) identified curcumin as its major component, attributing antibacterial, anti-inflammatory, antimicrobial, and anticancer properties to it (Hay et al., 2019). Further research into the less-studied components of turmeric could unlock its broader applications and improve its comprehensive utilization.

Phytochemicals have garnered significant attention in recent years due to their pharmacological potency, low toxicity, and diverse health benefits. Among these, essential oils, which are key phytochemicals derived from aromatic and medicinal plants, exhibit a range of bioactivities, including anti-inflammatory, antioxidant, and anticancer properties (Li et al., 2022). The chemical composition of plant essential oils is highly complex, comprising primarily terpenoids, aromatic compounds, aliphatic compounds, and sulfur-containing compounds. Factors such as geographical origin, harvest season, and extraction methods further influence their composition (Caputo et al., 2020; Zhang et al., 2019). Sensory evaluation is a direct and efficient approach to distinguish volatile organic compounds (VOCs), but its subjectivity and susceptibility to personal biases necessitate to personal biases necessitate the involvement of trained professionals for reliable data. Advances in instrumentation have facilitated the integration of professional sensory analysis with intelligent analytical tools, enhancing objectivity and scientific rigor in VOC characterization. Among these tools, headspace gas chromatography–mass spectrometry (HS-GC–MS) is widely employed for VOC analysis due to its precision and versatility (Liu et al., 2020). Emerging technologies, such as electronic noses (e-noses), replicate the human olfactory system through sensor arrays and pattern recognition algorithms, offering novel insights into VOC profiles (Zhao et al., 2017). Gas chromatography-ion mobility spectrometry (GC-IMS) is another highly sensitive method for detecting trace organic compounds (Karpas, 2013), while headspace gas chromatography-ion mobility spectrometry (HS-GC-IMS) combines high sensitivity with rapid, continuous injection capabilities and rapid analysis. Compared to GC–MS, GC-IMS provides faster and more convenient VOC analysis (Jy et al., 2021). The integration of these methods enables comprehensive and accurate elucidation of plant chemical components and their differential changes under various conditions. This study represents the first application of a combined approach involving e-nose, GC-IMS, and GC–MS to analyze the VOCs of turmeric, turmeric essential oil, and by-products of turmeric essential oil. By leveraging these advanced analytical techniques, we systematically compared VOC profiles from plant raw materials, essential oils, and their by-products.

Additionally, this study investigates the VOC composition, color characteristics, and sensory attributes of five turmeric samples, including TP, TEO extracted at 60 °C, 90 °C, and 120 °C, and the by-products from oil extraction. The findings aimed to optimize TEO extraction temperatures and promote the sustainable utilization of turmeric, minimizing waste and maximizing its industrial applications.

## 2 Materials and methods

### 2.1 Samples and reagents

Turmeric powder, turmeric essential oil by-products and turmeric essential oil obtained by molecular distillation at different temperatures were provided by Chen Guang Biotechnology Group Co., Ltd. (Hebei, China).

Dried turmeric (moisture content of 12.69 %) was ground into powder with a grinder, and turmeric powder was obtained through a 60-mesh sieve, turmeric powder was extracted to extract curcumin after by-products of turmeric essential oil, After extracting curcumin, turmeric essential oil was extracted from turmeric oil through molecular distillation under the conditions of main tank temperature of 60, 90, and 120 °C, distillation pressure of 8 Pa, and feed rate of 35 mL/min. Extraction of turmeric essential oil. This study shared five different turmeric samples, viz. turmeric powder (TP), the by-products of turmeric essential oil (TEO-BP), TEO obtained by molecular distillation at 60 °C, 90 °C, and 120 °C (Named as TEO-60, TEO-90 and TEO-120, respectively). A tetracarbon-9 ketone standard of gas phase ion migration spectroscopy was purchased from Baoding Jet Company (Hebei, China). Other reagents used in the test were all analytically pure. ABTS diamond salt is provided by Shanghai Yuanye Biotechnology Co., Ltd. 2,2-diphenyl-1-picrylhydrazyl are provided by Solarbio Technology Co., Ltd. The Total antioxidant capacity assessment kit (FRAP method) is provided by Nanjing Jiancheng Institute of Biotechnology. α-Glucosidase is provided by Shanghai Yuanye Biotechnology Co., Ltd. *P*-nitrophenyl-β-D-glucopyranoside is provided by Shanghai Yuanye Biotechnology Co., Ltd.

### 2.2 Color analysis

The surface color of turmeric samples was evaluated by a portable precision colorimeter (WSC-2B) (Shanghai Yidian Physical Optical Instrument Co. Ltd., China). In order to ensure the rigor of the color analysis test, This study only compared liquid samples of turmeric and then the color test was performed. Color values were expressed in chromatic coordinates L* (lightness), a* (redness/greenness), b* (yellowness/blueness) coordinates. The total change in color of the turmeric essential oil samples with reference to the TEO-BP was computed as (Shi et al., 2020):

(1) $$\mathrm{\Delta E}=\bar{{\left(L_{0}-L^{*}\right)}^{2}+{\left(a_{0}-a^{*}\right)}^{2}+{\left(b_{0}-b^{*}\right)}^{2}}$$

where, subscript “0” refers to the color values of the TEO-BP (control).

### 2.3 Sensory evaluation

Five turmeric samples were evaluated using quantitative descriptive analysis. According to GB/T 1629.1–2012 (Sensory analysis selection, training and Part 1 of the General Guidelines for Managers for Evaluators), selected evaluators were screened and trained as sensory evaluation team members. Ten members (5 males and 5 females) between the ages of 20 and 30 years were selected to form the official evaluation team through basic tests of sensory sensitivity and motivation to participate. Room temperature was maintained at 27 ± 2 °C and humidity at 55 ± 3 %. Five turmeric samples (0.1 g) were weighed in a test tube (Lazo et al., 2016). Set up three sets of repeated experiments for each sample. The samples were randomly coded with Arabic numerals and scored based on five sensory attributes. These five sensory properties included herbal fragrance, aromatic, turmeric aroma, pine fragrance, and pungent. The flavor intensity was on a 10-point scale, where 0 points meant no taste and 10 points meant the strongest taste. The data results were analyzed by analysis of variance (ANOVA)(Zhou et al., 2016). The panelists (5 men and 5 women) scored their own perception of the smell of different turmeric samples on a 10-point scale (0 points for completely unacceptable, 10 points for complete acceptance).

Prior to the publication of this study, written informed consent forms were obtained from all participants, and all experimental participants agreed to participate in sensory activities and use their information.

### 2.4 *E*-nose analysis

The PEN 3 E-nose (Airsense Analytics Co. Ltd., Schwerin, Germany) with 10 different metal oxide sensors were used for E-nose analysis. Each sensor had a corresponding sensitive substance as shown in Table S1. (Shen et al., 2021). The aroma characteristics of five samples were determined with an electronic nose. The turmeric powder sample (0.20 g) and the turmeric liquid sample (10 μL) were placed in a 10 mL headspace vial and sealed for testing (Each sample in triplicate). The samples to be tested were incubated for 1 min at 40 °C with a headspace analysis injection volume of 1500 μL; a collection delay of 210 s; and a syringe temperature of 50 °C, referring to the method of Duan Mengya et al. (Duan et al., 2021). The data were analyzed with reference to the measurement conditions using the E-nose software system.

### 2.5 HS-GC-IMS analysis

The GC-IMS (Floorspace®, G.A.S. Gesellschaft für Analytische Sensorsysteme mbH, Dortmund, Germany) instrument was equipped with a syringe and an automatic headspace sampling device. First of all, 0.5 g sample was accurately weighed into 20 mL headspace bottles, which was incubated at 40 °C at 250 r/min (Solid sample) and 500 r/min (Liquid sample) for 10 min. The extracted headspace air volume was 100 μL and the syringe temperature was 45 °C. The extracted VOCs were pre-isolated by gas chromatography columns, coupled with IMS. The column temperature was 60 °C and the carrier gas was composed of 99.99 % pure nitrogen (Yao et al., 2021). The carrier gas flow system initially had a flow rate of 1 mL/min; increased to 2 mL/min at the 2 min marker; increased to 10 mL/min at the 5 min marker; increased to 30 mL/min at the 10 min marker; increased to 50 mL/min at the 15 min marker; increased to 100 mL/min at the 20 min marker; and then maintained at the 30 min marker (Drabinska et al., 2018). The pre-separated compound was ionized by an ionization source (5.68 keV) in the IMS ionization chamber and then transferred to a 9.8 cm drift tube at 45 °C with a nitrogen gas flow rate of 150 mL/min (Mdmc et al., 2020). The VOCs were identified according to the RIs (Retention Indexes) of the reference materials in the GC-IMS library (G.A.S.) and the retention time and measurement method of the test determination. And the peak area normalization method was used to quantify the substances.

### 2.6 GC–MS analysis

Some improvements have been made in the extraction and analysis of VOCs based on published research articles. Weighed TP (30 mg) was mixed thoroughly with 1 mL ethanol by sonication for 10 min. After centrifugation, the supernatant was filtered through 0.22 μm microporous membrane filter and directly loaded for GC–MS analysis. Other liquid samples were diluted (10 times) using ethanol and filtered through 0.22 μm microporous membrane filter before loading for GC–MS analysis. In the 7890 A-5975C gas analyzer (Agilent Technology Co., Ltd., USA), the filtered sample was directly loaded for analysis. The VOCs were separated using a HP-5MS (30.0 m × 250 μm, 0.25 μm). The carrier gas was helium flowing at a rate of 1.0 mL/min. The starting temperature of the column was 50 °C; rising to 150 °C at a speed of 5 °C/min to maintain for 2 min; rising to 200 °C at a speed of 3 °C/min to maintain for 2 min; rising to 280 °C at a speed of 10 °C/min maintain for 2 min. The temperature of gasification chamber is 250 °C. The temperature of the transmission line is 250 °C. The ion source was operated in electron impact (EI) mode at 70 eV. The temperature of ion source is 230 °C, and the temperature of quadrupole is 150 °C. Scan mode was adopted across the *m*/*z* range of 20–500. Solvent delay was 3 min (Sharma et al., 2021).

The detected compounds were characterized by the MS database NIST11 using retention time, and retention index (Madhumita et al., 2019). The area normalization quorum, which was the percentage of the peak area of the identified component as the sum of the areas of all identified components, was used as a quantitative result.

### 2.7 Determination of antioxidant activity

#### 2.7.1 DPPH radical-scavenging

Under dark conditions, transfer 100 μL of different turmeric sample solutions and mix them evenly with 300 μL of 0.4 mmol/L DPPH anhydrous ethanol solution. Measure the absorbance value A_X_ at 517 nm after 30 min of reaction at room temperature. The formula for calculating DPPH radical scavenging rate was as follows:

(2) $$I_{\mathrm{DPPH}}=\left(1-\frac{A_{X}}{A_{0}}\right)\times 100\%$$

where，subscript “0” refers to the ethanol group (control).

#### 2.7.2 ABTS radical-scavenging

The 7 mmol/L ABTS aqueous solution was mixed with 2.45 mmol/L K2S2O8 aqueous solution in equal volume and allowed to stand for 16 h at room temperature protected from light. The absorbance of ABTS solution was diluted to 0.7 ± 0.02 using anhydrous ethanol, and set aside. The absorbance A_X_ of ABTS solution was measured at 734 nm after 10 min of mixing 100 μL of different turmeric samples with 1 mL of ABTS solution in a light-proof environment. The formula for calculating the radical scavenging rate of ABTS was as follows:

(3) $$I_{\mathrm{ABTS}}=\left(1-\frac{A_{X}}{A_{0}}\right)\times 100\%$$

where，subscript “0” refers to the ethanol group (control).

#### 2.7.3 Ferric reducing antioxidant power

Ferric reducing antioxidant power was determined using FRAP kit (Nanjing Jianjian Bioengineering Institute). 100 μL of different turmeric sample solutions were mixed with 3 mL of FRAP. The mixture was then allowed to stand for 5 min in a water bath at 37 °C. The absorbance of the sample was read at 593 nm. Blanks were prepared by replacing the samples with water. Different concentrations of FeSO_4_-7H_2_O were prepared to obtain the standard calibration curve. The chelating capacity was calculated as FRAP value (μmol/L Fe^2+^).

### 2.8 α-Glucosidase inhibitory activity

Phosphate buffer (pH = 6.8) was used to prepare 1 U/mL α-glucosidase solution and 2.5 mmol/L PNPG solution, respectively. 40 μL of α-glucosidase solution as well as different turmeric samples solution were added into 96-well petri dishes. The reaction was mixed and held at 37 °C for 15 min, then 20 μL of PNPG solution was added and mixed and held at 37 °C for 15 min. Finally, 150 μL of 0.2 mol/L Na2CO3 solution was added to stop the reaction, and the absorbance A_X_ at 405 nm was measured. The α-glucosidase inhibitory activity was calculated using the following formula:

(4) $$I_{\alpha -\text{glucosidase}}=\left(1-\frac{A_{X}}{A_{0}}\right)\times 100\%$$

where，subscript “0” refers to the ethanol group (control).

### 2.9 Data analysis

Principal component analysis (PCA) and radar plotting were performed using Origin 2019 (Origin Lab Corporation, Northampton, MA, USA). Histograms were produced by Chiplot (https://www.chiplot.online/). Five fingerprints were generated based on GC-IMS analysis. Each point in the plot represented a volatile substance and was characterized by retention time, relative drift time, and signal intensity. Analysis of variance (ANOVA) as well as discriminant analysis were done with SPSS.23. PCA and partial least squares discriminant analysis (PLS-DA) were used to analyss HS-GC–MS and HS-GC-IMS's results by using MetaboAnalyst 5.0.

## 3 Results and discussion

### 3.1 Color characteristics

**Fig. 1:**
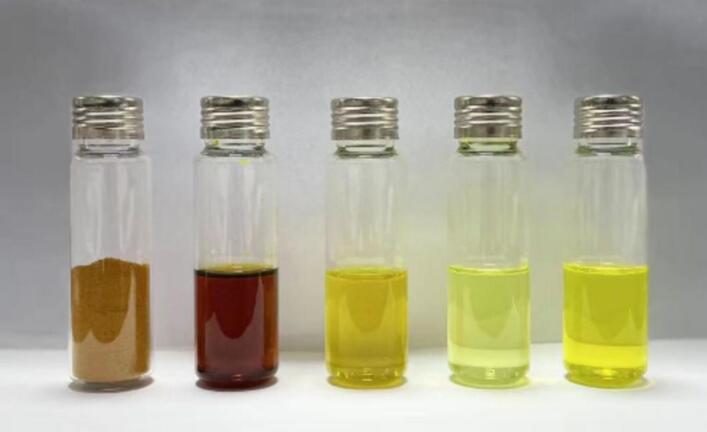
Five different turmeric samples (Order from left to right turmeric powder, the by-products of turmeric essential oil and three turmeric essential oil obtained by molecular distillation at 60 °C, 90 °C and 120 °C).

The color characteristics of the five turmeric samples are illustrated in Fig. 1. Color measurements were based on brightness (L*), red (a*), and yellow (b*), while the color difference (*ΔE*) was used to quantify variations in turmeric essential oil (TEO) samples produced by molecular distillation at different temperatures, relative to TEO-BP (Arslan & Özcan, 2008). Significant differences (*p* < 0.05) were observed among the samples in L*, a* and b* values. TEO samples exhibited higher L* values compared to TP, likely due to TEO’ s transparency, which enhances light transmission. In contrast, TEO-BP and TEO showed lower b* values than those of TP, reflecting TP's darker and more uniform coloration (Aksoy et al., 2019).

As shown in Table 1, TEO distilled at 60 °C had the highest *ΔE* value, indicating the greatest color difference from TEO-BP. Conversely, *ΔE* values for TEO obtained at 90 °C and 120 °C were relatively smaller. These findings suggest that molecular distillation induces changes in TEO color, characterized by increased L* values and decreased b* values, likely resulting from the volatilization and reaction of organic compounds during the distillation process. With increasing temperature, the total color difference (Δ*E*) decreased, indicating that molecular distillation at 90–120 °C had a minimal impact on TEO's major components, thereby stabilizing both color and composition. Furthermore, TEO yield remained consistent when the distillation temperature ranged between 100 °C and 140 °C (Huang et al., 2012). These observations identify the temperature range of 90–120 °C as optimal for producing TEO and related products. Given the darker color of TEO-BP, its use in food and cosmetic industries should be carefully controlled to avoid undesirable product coloration in final products.

**Table 1:** Color characteristic and sensory scores of five different turmeric samples.

Variables	TP	TEO-BP	TEO-60	TEO-90	TEO-120
Lightness (*L*^*⁎*^ value)	52.16 ± 0.99	50.46 ± 0.23^d^	61.89 ± 0.30^b^	60.44 ± 0.93^c^	62.89 ± 0.22^a^
Redness (*a*^*⁎*^ value)	12.43 ± 0.39	8.36 ± 0.09^a^	6.49 ± 0.03^b^	3.56 ± 0.09^d^	4.46 ± 0.07^c^
Yellowness (*b*^*⁎*^ value)	29.89 ± 0.44	12.81 ± 0.90^d^	25.17 ± 0.45^a^	15.41 ± 0.10^c^	19.26 ± 0.47^b^
Color difference (*ΔE*)	–	–	16.94 ± 0.22^a^	11.39 ± 0.83^c^	14.54 ± 0.05^b^
Herbal fragrance	9.10 ± 0.88^a^	7.60 ± 0.70^b^	7.80 ± 0.12^b^	5.30 ± 0.39^c^	3.60 ± 0.33^d^
Aromatic	5.10 ± 0.74^c^	6.00 ± 0.82^c^	4.90 ± 0.06^b^	6.20 ± 0.42^b^	7.50 ± 0.35^a^
Turmeric aroma	9.70 ± 0.48^a^	8.50 ± 0.53^b^	8.00 ± 0.03^b^	6.70 ± 0.27^c^	5.90 ± 0.23^d^
Pine fragrance	7.90 ± 0.74^a^	6.90 ± 0.57^b^	7.20 ± 0.12^b^	4.30 ± 0.32^c^	4.00 ± 0.27^c^
Pungent	5.50 ± 0.53^c^	7.20 ± 0.63^b^	8.60 ± 0.07^a^	4.30 ± 0.30^d^	3.40 ± 0.25^e^

### 3.2 Sensory evaluation analysis of turmeric samples

**Fig. 2:**
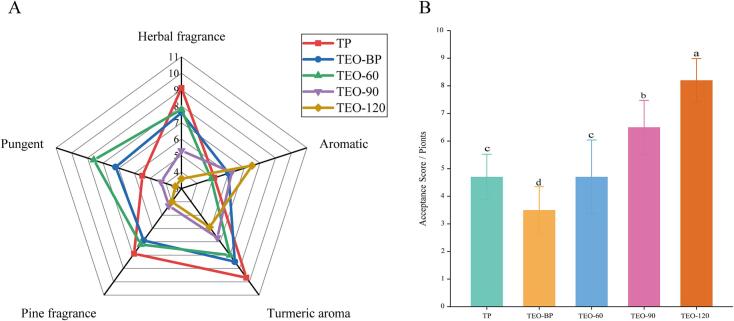
(A) Sensory evaluation radar of five different turmeric samples. (B) Histogram of sensory receptivity scoring of five turmeric samples.

The sensory evaluation analysis of different turmeric samples, illustrated in Fig. 2A, assessed five attributes: herbal fragrance, aromatic, turmeric aroma, pine fragrance, and pungent. Results summarized in Table 1 show that sample TP exhibited significantly higher herbal fragrance scores compared to the other samples (*p* < 0.05), while TEO-BP and TEO-60 showed no significant differences in herbal fragrance (*p* > 0.05). The herbal fragrance in these samples is likely due to volatile organic compounds, such as α-phellandrene and 1,8-cineole, both of which are associated with minty and herbal notes (discussed in Section 3.4).

**Fig. 3:**
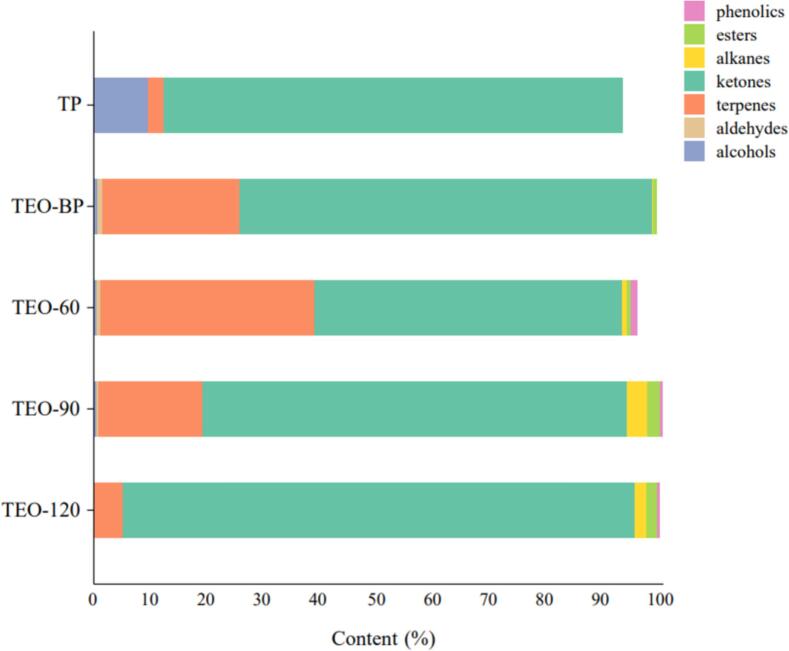
Columnar stacking of the relative content (%db.) of volatile organics in five turmeric samples.

The aromatic score of TEO-120 was significantly higher than the other samples (*p* < 0.05), while TP, TEO-BP, TEO-60, and TEO-90 showed no significant differences (*p* > 0.05). These differences likely result from variations in the composition of volatile substances. Similarly, TP displayed the strongest turmeric aroma (p < 0.05), while TEO-120 had the weakest. Interestingly, TEO-BP exhibited a significantly stronger turmeric aroma than the other liquid samples, highlighting its potential as a source of high-value components. The pine aroma score of TP was significantly higher than the other samples, mirroring the herbal fragrance trend. In contrast, TEO-120 exhibited significantly lower pungency than those of the other four samples (*p* < 0.05), likely due to the removal of aldehydes and phenols during high-temperature distillation, which generated new aromatic terpenoids and esters. As shown in Fig. 3, where the abundance of VOC species decreased with increasing distillation temperature, consistent with these findings.

Overall acceptability scores, depicted in Fig. 2B, ranked TEO-120 highest, followed by TEO-90, TEO-60, and TP, with TEO-60 ranking third, showing no significant difference in acceptability compared to TP. The superior acceptability of TEO-120 can be attributed to its higher aromatic scores and reduced pungency. These results suggest that further processing is necessary for TEO-BP, particularly to separate or degrade undesirable volatile components, ensuring its suitability for broader applications. VOCs in these samples were subsequently analyzed (Section 3.4) to establish a comprehensive database supporting the development and application of TEO-BP.

### 3.3 *E*-nose results

**Fig. 4:**
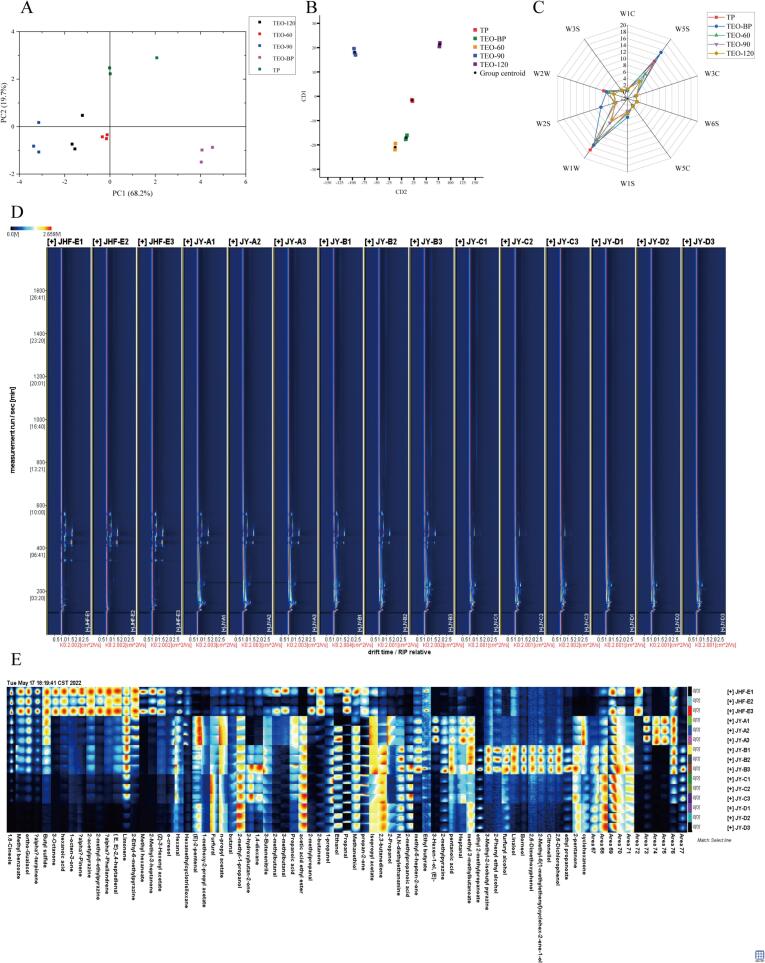
(A) Principal component analysis (PCA) of five turmeric samples. (B) Canonical discriminant analysis (CDA) of five turmeric samples. (C) Radar plots of electronic nose response data for five turmeric samples. (D) Two-dimensional chromatogram results of volatile fractional compositions in five turmeric samples. (E) The VOCs fingerprint of five turmeric samples. (JHF-E represents TP; JY-A represents TEO-BP; JY-B represents TEO-60; JY-C represents turmeric essential oil obtained by TEO-90; and JY-D represents TEO-120. In addition, the numbers 1, 2, 3 represent three parallels.)

The development of software-assisted computing has expanded possibilities in fields such as healthcare, Principal component analysis (PCA), a robust multivariate tool, is widely employed for processing and analyzing high-dimensional data in disciplines such as biology and chemistry (Falasconi et al., 2005). In this study, PCA was applied to distinguish among the five turmeric samples based on electronic nose data. As shown in Fig. 4A, the first two principal components, PC1 and PC2, accounted for 68.2 % and 19.7 % of the variance, respectively. The relative distances between the five samples, particularly the distinct separation of TEO-BP from the other four, indicated significant differences in VOC profiles.

Canonical discriminant analysis (CDA), another multivariate method, corroborated these findings. As depicted in Fig. 4B, CDA effectively separated the samples using two discriminant functions, demonstrating the utility of combining PCA and CDA for distinguishing turmeric samples via electronic nose data.

The radar plot (Fig. 4C) revealed that sensors W2W, W1W and W5S showed strong responses to VOCs, suggesting high levels of aromatic compounds, terpenes, ketones and nitrogen oxides in the samples. Notably, TEO-BP elicited a pronounced response from the W2S sensor, indicative of elevated concentrations of alcohols, aldehydes, and ketones. These findings align with TEO-BP's low sensory acceptability and unpleasant odor, distinguishing its odor profile from the other samples. Overall, the electronic nose analysis demonstrated that VOCs in TEO and its raw materials were predominantly terpenes, nitrogen oxides and aromatic compounds. However, the electronic nose exhibited limited sensitivity to certain VOCs. For instance, it could not detect alcohol-smelling substances in Zanthoxylum bungeanum Maxim (Xfab et al., 2021). Consequently, while the electronic nose identified key aroma characteristics of turmeric samples can be identified using the e-nose. More detailed analysis of the five turmeric samples, comprehensive analysis requires integration with additional techniques for VOC profiling.

### 3.4 Identification of VOCs using HS-GC-IMS

HS-GC-IMS, an advancement of ion mobility spectrometry (IMS), was originally developed in the 1960s as a gas-phase separation technique for detecting and characterizing VOCs based on their ion mobility in the gas phase (Denawaka et al., 2014; Wang et al., 2020). In this study, GC-IMS was employed to analyze VOCs in various turmeric samples. Unlike GC–MS, which identifies analytes based on chemical morphology, GC-IMS employs two-dimensional (2D) separation using retention time and drift time. As shown in the Fig. 4D, the horizontal axis represents drift time, while the vertical axis denotes retention time. The red vertical line on the left marks the active ion peak, while the background is depicted in blue. Each point in the Fig. 4E corresponds to a specific VOC, with hues indicating concentration levels: white dots represent lower concentrations, red dots signify higher concentrations, and darker hues denote increasing concentrations. VOCs were identified using the NIST gallery, considering retention index (RI), retention time, drift time, and kovts (Ko). A total of 77 peaks were detected, representing 65 distinct VOCs, including 13 alcohols, 11 ketones, 10 esters, 10 aldehydes, 8 phenols and acids, 7 hydrocarbons, and 6 miscellaneous compounds; 12 peaks remained unidentified.

The primary VOCs in the turmeric samples included terpenoids such as limonene, alpha-phellandrene, α-pinene, and 1,8-cineole, consistent with previous studies. Relative concentrations were visualized through fingerprint intensities, with darker hues indicating higher levels. In TP, abundant VOCs included 1,8-cineole, methyl benzoate, ortho-guaiacol, alpha-terpinene, butyl sulfide, 3-octanone, α-pinene, 2-methyl-5-ethylpyrazine, limonene, methyl hexanoate, methanethiol, and propan-2-one. Several VOCs, such as 1,8-cineole, methyl benzoate, ortho-guaiacol, alpha-terpinene, and butyl sulfide, exhibited decreasing intensities with rising molecular distillation temperatures, likely due to thermal degradation (Pirbalouti et al., 2013). Conversely, VOCs, such as acetic acid ethyl ester, 2-methylpropanoic acid were less abundant in TP but more prevalent in TEO-BP and TEO. For example, TEO-BP contained VOCs like butyral and *o*-methyl phenol, contributing to its low sensory acceptability due to their strong, unpleasant odors. Some VOCs, including furfural and acetic acid ethyl ester, increased with higher distillation temperatures, possibly due to esterification reactions between acids and alcohols (Díaz-Maroto et al., 2004). Similarly, α-pinene's conversion to linalool might explain its decreasing content, as both thermal decomposition and chemical transformation occurred (Hamrouni-Sellami et al., 2013). Volatile compounds such as linalool and borneol were most abundant in TEO-60, likely because the lower distillation temperature preserved these compounds without significant degradation or loss. Additionally, some unidentified VOCs displayed minimal sensitivity to distillation temperature changes, suggesting their relative stability under varying thermal conditions. This analysis underscores the complex interplay between molecular distillation temperature and VOC composition, providing insight into the thermal stability and transformation of key aroma compounds in turmeric samples.

### 3.5 VOC characterization using GC–MS

The VOCs of five turmeric samples were analyzed using GC–MS. To enhance clarity, bar graphs illustrating the volatile organic compounds and a table summarizing their relative contents were prepared. As shown in Table 2 and Fig. S1, 62 compounds were identified, including 28 terpenes, 5 alcohols, 8 ketones, 3 esters, 12 hydrocarbons, 2 aldehydes and 3 phenols. Among these, key turmeric sample contained the most turmeric flavonoids such as ar-turmerone, turmerone, zingiberene, and α-curcumene were consistently present across all samples. Fig. 3 highlights the predominance of ketones, which constituted more than half of the total VOCs in each sample. In turmeric powder samples, ketones dominated, accompanied by smaller proportions of alcohols. Liquid turmeric samples, in contrast, contained higher terpene levels in addition to ketones. Notably, the relative content of ar-turmerone was significantly higher in the liquid turmeric samples compared to the turmeric powder (Table 2). This increase could be attributed to two factors: (1) volatilization of other organic compounds in turmeric essential oil, which proportionally elevated ar-turmerone content, or (2) oxidative rearrangement of unstable ar-turmerone and β- turmerone into a more stable ar-turmerone structure (Nair, 2013). In both scenarios, the increased relative ar-turmerone content resulted from elevated temperatures during TEO extraction.

**Table 2:** Volatile organic compound composition and relative content tables of five different turmeric samples.

S.NO	Compounds	Molecular formula	Relative content of components (Peak area, %)				
			TP	TEO-BP	TEO-60	TEO-90	TEO-120
1	α-phellanderene	C_10_H_16_	0.27±0.06^a^	0.06±0.00^b^	0.02±0.01^b^	-	-
2	p-Cymene	C_10_H_14_	-	0.03±0.01^a^	0.09±0.00^a^	-	-
3	1,8-Cineole	C_10_H_18_O	0.13±0.04^b^	0.24±0.00^a^	0.15±0.02^b^	0.01±0.00^c^	-
4	Terpinen-4-ol	C_10_H_18_O	-	0.01±0.00^a^	0.05±0.00^a^	-	-
5	(-)-Cedrene	C_15_H_24_	-	0.00±0.00^c^	0.13±0.01^a^	0.01±0.00^b^	0.07±0.00^b^
6	β-Terpinene	C_10_H_16_	-	0.04±0.01	-	-	-
7	Santalene	C_15_H_24_	-	2.11±0.02	-	-	-
8	α-Bergamotene	C_15_H_24_	-	0.34±0.00^a^	0.98±0.62^ab^	0.04±0.00^b^	-
9	Humulene	C_15_H_24_	-	0.07±0.00^a^	0.28±0.00^a^	0.06±0.00^a^	-
10	cis-β-Farnesene	C_15_H_24_	-	0.47±0.01^a^	0.80±0.00^a^	0.20±0.00^a^	
11	β-Himachalene	C_15_H_24_	-	0.19±0.00^a^	0.16±0.00^a^	-	-
12	α-Curcumene	C_15_H_22_	0.73±0.17^c^	4.92±0.05^b^	9.56±0.49^a^	4.66±0.14^b^	0.71±0.03^c^
13	Zingiberene	C_15_H_24_	0.91±0.26^c^	5.00±0.05^b^	10.20±0.87^a^	4.88±0.15^b^	0.92±0.04^c^
14	3 Oxabicyclo[6.3.1]dodec-8-en-2-one	C_11_H_16_O_2_	-	0.02±0.00^d^	1.16±0.02^a^	0.82±0.04^b^	0.23±0.00^c^
15	β-Bisabolene	C_15_H_24_	-	1.89±0.02^a^	2.19±0.24^a^	-	-
16	β-Curcumene	C_15_H_24_	-	0.24±0.01^c^	0.80±0.03^a^	0.48±0.08^b^	0.15±0.15^c^
17	β-Sesquiphellandrene	C_15_H_24_	0.83±0.28^d^	5.76±0.05^b^	8.55±0.67^a^	4.77±0.14^c^	1.09±0.05^d^
18	α-sinensal	C15H_22_O	-	0.30±0.00^c^	0.68±0.00^a^	0.33±0.01^b^	-
19	(+)-2-Carene	C_10_H_16_	-	0.02±0.00	-	-	-
20	(+)-Camphene	C_10_H_16_	-	0.10±0.00^a^	-	-	0.01±0.00^a^
21	3,4,4-trimethyl-cyclohex-2-enone	C_9_H_14_O	-	0.16±0.01^b^	0.37±0.01^a^	0.29±0.01^a^	0.09±0.01^c^
22	3,3,5,5-Tetramethylcyclopentene	C_9_H_16_	-	0.13±0.01^c^	0.38±0.00^a^	0.31±0.00^b^	0.11±0.00^c^
23	2,4,4,6-Tetramethyl-6-phenyl-1-heptene	C_17_H_26_	-	1.76±0.01^a^	0.18±0.00^c^	-	0.25±0.00^b^
24	3- Methyl-2-butenoic acid, 4- 2,7-dimethyloct-7-en-5-yn-4-yl ester	C_15_H_22_O_2_	-	0.32±0.34^c^	0.18±0.01^c^	0.59±0.05^b^	1.36±0.10^a^
25	(Z)-citral	C_10_H_16_O	-	0.51±0.02^a^	0.17±0.00^b^	-	-
26	Ar-tumerone	C_15_H_20_O	23.21±1.42^d^	44.40±0.56^b^	34.50±0.74^b^	45.99±2.04^c^	54.56±0.67^a^
27	Tumerone	C_15_H_22_O	36.87±2.66^a^	5.50±0.01^b^	5.57±0.94^b^	6.33±0.31^b^	7.11±0.28^b^
28	α-Bisabolol	C_15_H_26_O	-	0.26±0.01^a^	0.08±0.02^c^	0.20±0.00^b^	-
29	Curlone	C_15_H_22_O	20.56±1.50^b^	22.23±0.25^b^	12.34±0.77^c^	20.98±0.62^b^	27.91±1.22^a^
30	Benzene, 1,2,3,5-tetramethyl-	C_10_H_14_	-	1.02±0.02^a^	0.34±0.00^a^	1.00±0.06^a^	1.30±1.65^a^
31	Squalene	C_30_H_50_	-	0.01±0.00	-	-	-
32	Terpinolene	C_10_H_16_	-	-	0.03±0.03	-	-
33	Piperitone	C_10_H_16_O	-	-	0.01±0.00	-	-
34	Isoeugenol	C_10_H_12_O_2_	-	-	0.01±0.00	-	-
35	(-)-α-Copaene	C_15_H_24_	-	-	0.29±0.40	-	-
36	(+)-4-Carene	C_10_H_16_	-	-	0.11±0.00	-	-
37	Caryophyllene	C_15_H_24_	-	-	2.39±0.00^a^	0.44±0.01^b^	0.01±0.00^c^
38	α-ylangene	C_15_H_24_	-	-	0.32±0.29	-	-
39	β-Santalol	C_15_H_24_O	-	-	0.36±0.00^a^	-	0.11±0.00^a^
40	Heptacosane	C_27_H_56_	-	-	0.00±0.00^a^	0.00±0.00^a^	0.00±0.00^a^
41	Docosane	C_22_H_46_	-	-	0.00±0.00^a^	-	0.00±0.00^a^
42	Hexacosane	C_26_H_54_	-	-	0.01±0.00^a^	-	0.00±0.00^a^
43	Heneicosane	C_21_H_44_	-	-	0.00±0.00^b^	0.01±0.00^a^	-
44	3,11-Acoradiene	C_15_H_24_	-	0.01±0.00^a^	0.71±0.77^a^	0.28±0.25^a^	-
45	(E)-β-farnesene	C_15_H_24_	-	-	-	1.08±0.03^a^	0.19±0.01^b^
46	Ethanone, 1-(1,3-dimethyl-3-cyclohexen-1-yl)-	C_10_H_16_O	-	-	0.09±0.00^a^	0.06±0.00^b^	-
47	1-(1,2,3-Trimethyl-cyclopent-2-enyl)-ethanone	C_9_H_16_O	-	-	0.09±0.00^c^	0.30±0.00^a^	0.12±0.00^b^
48	Ethanone, 1-(1,4-dimethyl-3-cyclohexen-1-yl)	C_10_H_16_O	-	-	-	0.06±0.00^a^	0.01±0.00^b^
49	1,2-Benzenediol, o-(2-bromopropionyl)-o'-(3-methylbenzoyl)	C_17_H_15_BrO_4_	-	0.22±0.03^a^	-	0.33±0.00^a^	-
50	Benzene, 1-(3-cyclopentylpropyl)-2,4-dimethyl	C_16_H_24_	-	-	-	2.70±0.09^a^	1.93±0.00^a^
51	α-Santalol	C_15_H_24_O	-	-	0.43±0.00^b^	0.50±0.00^a^	0.36±0.00^c^
52	2,4-Dimethylpentan-3-yl (E)-2-methylbut-2-enoate	C_12_H_22_O_2_	-	-	0.03±0.00^b^	0.20±0.15^b^	0.40±0.10^a^
53	Prop-2-ynyl (E)-2-methylbut-2-enoate	C_8_H_10_O_2_	-	0.31±0.01^a^	0.35±0.09^a^	1.37±2.14^a^	0.12±0.00^a^
54	Tetracosane	C_24_H_50_	-	-	0.00±0.00^a^	0.01±0.00^a^	-
55	1-Iodotridecane	C_13_H_27_I	-	-	0.01±0.00^a^	0.01±0.00^b^	0.00±0.00^c^
56	Di-epi-α-cedrene-(I)	C_15_H_24_	-	0.07±0.11^a^	-	0.16±0.08^a^	0.05±0.00^a^
57	Aromadendrene, dehydro-	C_15_H_22_	-	-	-	-	0.01±0.00
58	1,6-Cyclodecadiene, 1-methyl-5-methylene-8-(1-methylethyl)-, [S-(E,E)]-	C_15_H_24_	-	0.06±0.00^c^	0.08±0.00^b^	0.11±0.00^a^	0.08±0.01^c^
59	2-Nonadecanone	C_19_H_38_O	-	0.02±0.00^a^	-	-	0.11±0.00^a^
60	Eicosane, 10-methyl-	C_21_H_44_	-	-	0.01±0.00^a^	0.00±0.00^b^	0.00±0.00^b^
61	o-Cymene	C_10_H_14_		-	0.05±0.00^b^	0.33±0.00a	-
62	stigmasterol	C_29_H_48_O	9.46±0.00^a^	0.09±0.07^b^	-	-	-

Note: “-” - not detected;

Values within a row with the same letters were not significantly different (*p* < 0.05). Each value is the mean ± standard deviation of three replications.

Table 2 further reveals that the major components of TP, TEO-BP, and TEO (listed in descending abundance) included ar-turmerone, turmerone, zingiberene, α-curcumene, β-sesquiphellandrene, and β-bisabolene. Importantly, the composition of these key components remained consistent across samples, even when the distillation temperature exceeded 60 °C, with only their relative contents varying. Additionally, as the molecular distillation temperature increased, its impact on the species of major components diminished, though no linear trend of increasing or decreasing content with temperature was observed. Molecular distillation at approximately 90 °C was found to be optimal for extracting TEO. This temperature not only preserved the major components but also minimized energy consumption, particularly heating, thereby improving efficiency. TEO-BP exhibited the highest VOC diversity among the samples and contained elevated levels of bioactive compounds such as ar-turmerone and turmerone, underscoring its significant potential for application. The results emphasize the urgent need to explore and develop TEO-BP for its high-value components.

### 3.6 Comparison of abilities of GC–MS and GC-IMS to identify VOCs of turmeric samples

**Fig. 5:**
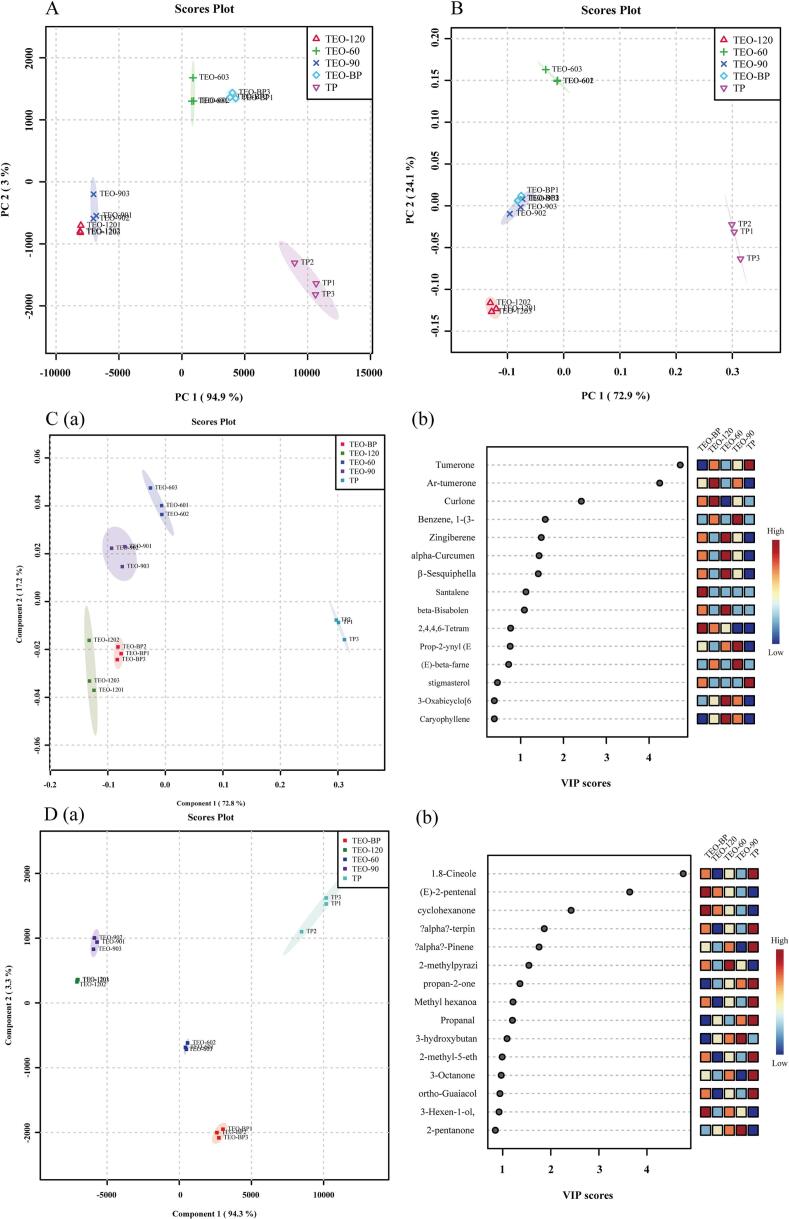
(A) Principal component analysis (PCA) scores plot of VOCs absolute content detected by HS-GC–MS of five turmeric samples. (B) PCA scores plot of VOCs intensity detected by HS-GC-IMS. Partial least squares-discriminant analysis (PLS-DA) of (C) Gas chromatography-mass spectrometry (HS-GC–MS) data and (D) gas chromatography-ion mobility spectrometry (HS-GC-IMS) data. (a) PLS-DA score plot of five turmeric samples. (b) Variable importance in projection (VIP) scores and cross-validation results of five turmeric samples.

GC–MS and GC-IMS were employed to identify and analyze the volatile compounds in turmeric samples, with GC–MS also quantifying their concentrations. PCA was used to visualize the data and differentiate the samples (Chai et al., 2023). While GC-IMS excels in rapid screening and real-time detection, GC–MS is more effective for detailed characterization and analysis of complex samples. The integration of both techniques leverages their complementary strengths, providing a comprehensive analysis of VOCs. As shown in Fig. 4 and Table 2, VOCs detected by HS-GC–MS in turmeric samples generally had higher boiling points than those identified by HS-GC-IMS, which demonstrated greater sensitivity to high-boiling-point VOCs. To further explore VOC variations among samples and their transformation from TP to TEO, PCA analysis was conducted based on data from both techniques, as shown in Fig. 5.

The PCA scores from GC–MS data (Fig. 5A) revealed that PC1 and PC2 accounted for 72.9 % and 24.1 % of the total variance, respectively. The close proximity of TEO-BP to TEO-90 in this plot suggested similar aromatic profiles, likely reflecting comparable VOC compositions. This finding aligns with the colorimetric analysis of the samples. Similarly, the PCA scores from GC-IMS data (Fig. 5B) showed that the first two principal components captured 97.9 % of the total variance. In this plot, TEO-BP clustered near TEO-60, and TEO-90 was closer to TEO-120, indicating comparable aroma compositions detected by GC-IMS. These results were consistent with sensory evaluations. Notably, TP was positioned farther from the other four samples in both PCA plots, highlighting distinct differences in VOC profiles between TP and the liquid samples. Additionally, GC-IMS demonstrated superior capability in distinguishing among closely related turmeric samples compared to GC–MS, as evident from the PCA clustering patterns.

PCA is an unsupervised dimensionality reduction technique well-suited for high-dimensional data. Partial least squares discriminant analysis (PLS-DA) combines orthogonal signal correction with PLS to identify differences in VOCs between turmeric samples analyzed via GC–MS or GC-IMS (He et al., 2021). Fifteen samples (five groups, three replicates each) were evaluated using PLS-DA, and the variable importance in projection (VIP) method was applied to identify characteristic aroma compounds for each sample across both models. The PLS-DA results are presented in Fig. 5. The PLS-DA model for GC–MS (Fig. 5C-a), based on 62 VOCs, accounted for 72.8 % and 17.2 % of the variance in the first and second components, respectively. Similarly, the GC-IMS PLS-DA model (Fig. 5D-a), using 65 VOCs, captured 94.3 % and 3.3 % of the variance in the first two components, respectively. Both models effectively distinguished the five groups of turmeric samples, as evidenced by the clear separation in the PLS-DA plots. Consistent with PCA results, the relative distance between sample positions in the PLS-DA plots indicated the degree of variation, with larger distances reflecting greater differences.

PLS-DA analysis was conducted using SIMCA 14.1.0 software to evaluate common aroma components and generate VIP values (Lin et al., 2024). The VIP score is a critical metric for assessing the impact of specific VOCs on distinguishing turmeric samples (Scavarda et al., 2020). Higher VIP values indicate greater contributions of VOCs to differences between samples and their significance in differentiating among turmeric groups.

A study compared the VOCs in lavender essential oil using GC-IMS and GC–MS, detecting 61 VOCs with GC-IMS and 84 with GC–MS. GC-IMS primarily identified terpenes (γ-pinene, camphorene, β-pinene), alcohols (linalool, (E)-2-octen-1-ol), and lipid compounds (linalyl acetate, butyl propionate, butyl acetate), while GC–MS predominantly detected esters (over 40 %), followed by alcohols, with terpenes, heterocycles, ketones, and aldehydes comprising about 20 %. Notably, GC-IMS identified certain compounds absent in GC–MS, such as alcohols (tetrahydro-linalool, isobutanol), esters (butyl propionate, propyl propionate, ethyl phenylacetate), and ketones (3-methyl-2-pentanone, methyl heptenone). This underscores GC-IMS's suitability for detecting small-molecule, low-abundance VOCs (Lin et al., 2024). Consistent with this study, GC-IMS detected 66 compounds, while GC–MS identified 62. In the GC–MS VIP chart (Fig. 5C-b), ten VOCs, including tumerone, ar-tumerone, curlone, 1-(3-cyclopentylpropyl)-2,4-dimethylbenzene, and zingiberene, had VIP values >1, indicating their importance in the PLS-DA model. Tumerone and ar-tumerone, with VIP values >4, were the most critical. Similarly, the GC-IMS VIP chart (Fig. 5D-b) highlighted ten key VOCs, such as 1,8-cineole, (E)-2-pentenal, and cyclohexanone, with 1,8-cineole and (E)-2-pentenal being the most significant. Overall, the VOC profiles of turmeric samples (TP, TEO-BP, TEO-60, TEO-90 and TEO-120) varied, as identified by both GC–MS and GC-IMS, which detected ten key differentiating VOCs. Combining VOC analysis with sample color and sensory attributes provides a more comprehensive understanding of VOC changes in turmeric during processing. This study addresses the limited research on the effects of distillation temperature on VOCs in vegetable-based essential oils, offering a theoretical foundation and data to improve turmeric processing techniques and optimize product utilization.

### 3.7 Comparison of antioxidant activity

**Fig. 6:**
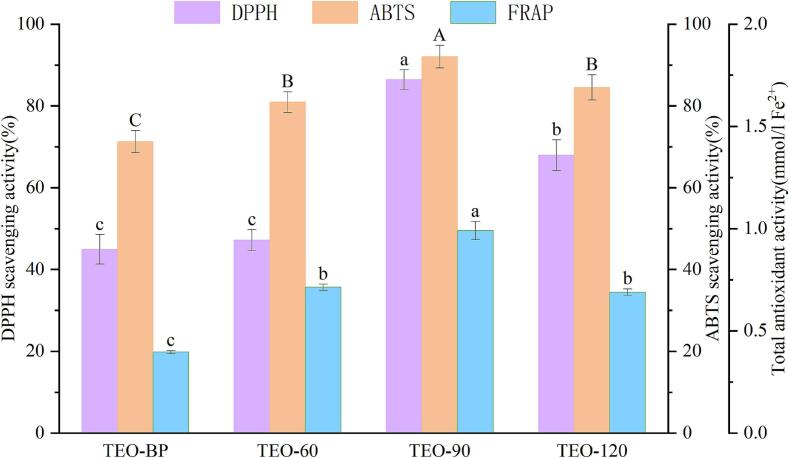
Determination of DPPH, ABTS free radical antioxidant activity, and total antioxidant capacity of four turmeric liquid samples.

Clearing free radicals is a key indicator of the body's ability to combat oxidative stress. The antioxidant activity of the four turmeric liquid samples was evaluated using ABTS and DPPH free radical scavenging assays, as well as the total antioxidant capacity assay (FRAP). As shown in Fig. 6, three of the turmeric liquid samples subjected to molecular distillation demonstrated superior antioxidant effects compared to TEO-BP. Among these, TEO-90 exhibited the highest antioxidant activity, scavenging 92.07 % of ABTS radicals. Its FRAP value was also the highest, indicating significantly stronger antioxidant effects than the other samples. The enhanced antioxidant capacity of TEO-90 can be attributed to its elevated levels of components such as 1,8-cineole and (−)-cedrene, which possess notable antioxidant properties. This finding highlights the potential therapeutic value of turmeric liquid samples and provides a basis for further research into their clinical applications.

### 3.8 Comparison of α-glucosidase inhibition

**Fig. 7:**
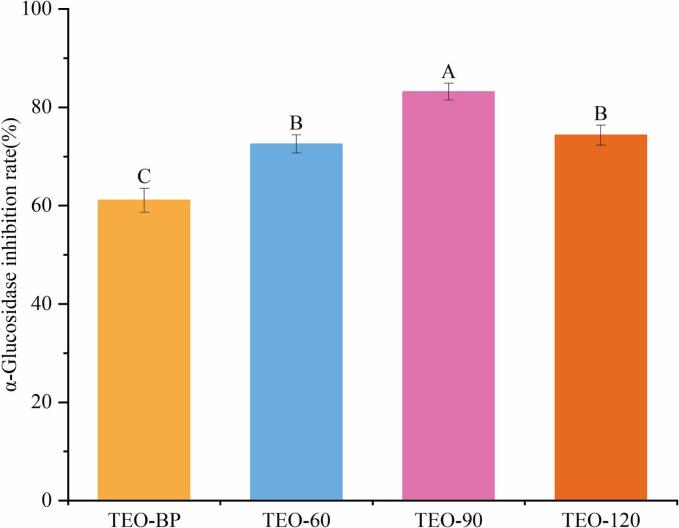
Inhibition of α-glucosidase by four turmeric liquid samples at the same concentration.

Alpha-glucosidase, an enzyme located in the small intestine, catalyzes the hydrolysis of oligosaccharides and polysaccharides in the intestines into absorbable monosaccharides. The use of appropriate α-glucosidase inhibitors can significantly reduce the postprandial blood glucose levels in diabetic patients. As shown in Fig. 7, all four turmeric liquid samples demonstrated α-glucosidase inhibitory activity, with three molecularly distilled samples exhibiting significantly stronger inhibition compared to TEO-BP. Among them, TEO-90 achieved the highest inhibition rate of 83.18 %, surpassing TEO-BP (61.10 %), TEO-60 (72.55 %) and TEO-120 (74.33 %). at the same concentration. The enhanced inhibitory effect of TEO-90 may be linked to its higher concentrations of active compounds capable of suppressing α-glucosidase, similar to its superior antioxidant activity. However, the precise mechanisms underlying this activity require further investigation. These results indicate that turmeric liquid samples, particularly TEO-90, can effectively inhibit α-glucosidase, potentially delaying carbohydrate absorption and aiding in blood glucose regulation.

## 4 Conclusion

This study characterized the physical and chemical properties of TP, TEO-BP, and TEO through color analysis and sensory evaluation. The VOCs in five turmeric samples were analyzed using electronic nose, GC–MS, and GC-IMS. Additionally, the in vitro antioxidant and α-glucosidase inhibitory activities of TEO-BP and TEO were investigated. Color analysis revealed that molecular distillation temperature influenced the color of TEO, likely due to two factors: the VOC composition of turmeric samples and reactions among organic substances induced by distillation temperature.

Sensory evaluation indicated a preference for samples with herbal and aromatic odors, suggesting these profiles enhance palatability. Monoterpenes and sesquiterpenes were identified as the dominant VOCs across samples using GC–MS, GC-IMS, and electronic nose analyses. The electronic nose effectively differentiated the five different turmeric samples based on flavor variations, with VOC variability observed during the transition from turmeric to TEO. A total of 77 compounds were detected by GC-IMS (12 unidentified) and 62 by GC–MS. The optimal molecular distillation temperature for TEO was determined to be approximately 90 °C, based on the concentration of key components. PCA and PLS-DA identified 20 key flavor compounds, including tumerone, ar-tumerone, curlone, 1,8-cineole, (E)-2-pentenal, and cyclohexanone, which effectively distinguished the five turmeric samples based on their VIP scores.

In vitro assays demonstrated that TEO-BP distilled at varying temperatures was enriched with antioxidant and α-glucosidase inhibitory compounds. Molecularly distilled turmeric samples showed enhanced bioactivities, with TEO-90 exhibiting the strongest antioxidant and α-glucosidase inhibition. This study provides valuable technical support and a theoretical framework for improving traditional turmeric processing and promoting comprehensive utilization. Further analysis of TEO-BP is recommended to optimize its development, minimize resource waste, and enhance its practical applications.

## CRediT authorship contribution statement

**Bing Yang:** Writing – review & editing, Supervision, Resources, Project administration, Methodology, Investigation, Funding acquisition, Data curation, Conceptualization. **Wanjia Wang:** Writing – review & editing, Writing – original draft, Visualization, Validation, Software, Resources, Project administration, Methodology, Investigation, Formal analysis, Data curation, Conceptualization. **Jianuo Zhang:** Writing – original draft, Validation, Software, Resources, Project administration, Formal analysis, Data curation, Conceptualization. **Wei Gao:** Visualization, Supervision, Resources, Funding acquisition, Conceptualization. **Lipeng Fan:** Visualization, Project administration, Funding acquisition, Data curation. **Bimal Chitrakar:** Writing – review & editing, Visualization, Validation, Supervision, Resources, Methodology, Investigation, Funding acquisition. **Yaxin Sang:** Writing – review & editing, Supervision, Resources, Project administration, Methodology, Investigation, Conceptualization.

## Declaration of competing interest

We declare that we do not have any commercial or associative interest that represents a conflict of interest in connection with the work submitted.

## Data Availability

Data will be made available on request.

## Appendix A Supplementary data

Supplementary material (Table S1 and Figure S1) Image 1

## References

[bb0005] Aggarwal B., Yuan W., Li S., Gupta S. (2013). Curcumin-free turmeric exhibits anti-inflammatory and anticancer activities: Identification of novel components of turmeric. Molecular Nutrition & Food Research.

[bb0010] Aksoy A., Karasu S., Akcicek A., Kayacan S. (2019). Effects of different drying methods on drying kinetics, microstructure, color, and the rehydration ratio of minced meat. Foods.

[bb0015] Arslan D., Özcan M. (2008). Evaluation of drying methods with respect to drying kinetics,mineral content and colour characteristics of rosemary leaves. Energy Conversion and Management.

[bb0020] Caputo L., Smeriglio A., Trombetta D., Cornara L., Trevena G., Valussi, M.,…Nazzaro, F. (2020). Chemical composition and biological activities of the essential oils of *Leptospermum petersonii* and *Eucalyptus gunnii*. Frontiers in Microbiology.

[bb0025] Chai X., Huang X., Zhang T., Wu K., Duan X., Yu H., Liu X. (2023). Comparative study of E-nose, GC-MS, and GC-IMS to distinguish star Anise essential oil extracted using different extraction methods. Separations.

[bb0030] Denawaka C., Fowlis I., Dean J. (2014). Evaluation and application of static headspace–multicapillary column-gas chromatography–ion mobility spectrometry for complex sample analysis. Journal of Chromatography A.

[bb0035] Díaz-Maroto M., Palomo E., Castro L., Viñas M., Pérez-Coello M. (2004). Changes produced in the aroma compounds and structural integrity of basil (*Ocimum basilicum* L) during drying. Journal of the Science of Food and Agriculture.

[bb0040] Drabinska N., Azeem H., Krupa-Kozak U. (2018). A targeted metabolomic protocol for quantitative analysis of volatile organic compounds in urine of children with celiac disease. RSC Advances.

[bb0045] Duan M.Y., Wang F.Q., Wu G.P., Li X., Gu F.L., Lin Y., Hou M.F. (2021). Analysis of flavor characteristics of four pepper essential oils. Food Science.

[bb0050] Falasconi M., Pardo M., Sberveglieri G., Ricco I., Bresciani A. (2005). The novel EOS835 electronic nose and data analysis for evaluating coffee ripening. Sensors and Actuators B: Chemical.

[bb0055] Guo T., Hao Q., Nan Z., Wei C., Liu J., Huang F., Wan C. (2022). Green extraction and separation of *Dendranthema indicum* essential oil by supercritical carbon dioxide extraction combined with molecular distillation. Journal of Cleaner Production.

[bb0060] Hamrouni-Sellami I., Rahali F., Rebey I., Bourgou S., Limam F., Marzouk B. (2013). Total Phenolics, flavonoids, and antioxidant activity of sage (*Salvia officinalis* L.) plants as affected by different drying methods. Food and Bioprocess Technology.

[bb0065] Hay E., Lucariello A., Contieri M., Esposito T., Perna A. (2019). Therapeutic effects of turmeric in several diseases: An overview. Chemico-Biological Interactions.

[bb0070] He X., Huang Y., Górska-Horczyczak E., Wierzbicka A., Jelen H. (2021). Rapid analysis of baijiu volatile compounds fingerprint for their aroma and regional origin authenticity assessment. Food Chemistry.

[bb0075] Huang H.F., Chen Y.X., Liang L.J., Wen-Pan S.U., Ping L.V., Feng C.M. (2012). Yield of molecular distillation turmeric oil under different temperature conditions and its components analysis by GC-MS. Science and Technology of Food Industry.

[bb0080] Jayaprakasha G.K., Negi P.S., Anandharamakrishnan C., Sakariah K.K. (2014). Chemical composition of turmeric oil--a byproduct from turmeric oleoresin industry and its inhibitory activity against different fungi. Zeitschrift Fur Naturforschung Section C-a Journal of Biosciences.

[bb0085] Jy A., Mw A., Rl A., Xiang L.A., Hui D.A., Lh, B.,…Hq, C. (2021). Application and development trends of gas chromatography–ion mobility spectrometry for traditional Chinese medicine, clinical, food and environmental analysis. Microchemical Journal.

[bb0090] Karpas Z. (2013). Applications of ion mobility spectrometry (IMS) in the field of foodomics. Food Research International.

[bb0095] Kumar G.S., Subramanian R. (2020). Recovery of bioactive volatiles from byproduct of curcumin manufacture by membrane processing. Industrial Crops and Products.

[bb0100] Kuttan R., Liju V., Jeena K. (2011). An evaluation of antioxidant, anti-inflammatory, and antinociceptive activities of essential oil from *Curcuma longa*. L. Indian Journal of Pharmacology.

[bb0105] Lazo O., Claret A., Guerrero L. (2016). A comparison of two methods for generating descriptive attributes with trained assessors: Check-all-that-apply (CATA) vs. free choice profiling (FCP). Journal of Sensory Studies.

[bb0110] Li Y., Cao X., Sun J., Zhang W., Zhang J., Ding Y., Liu Y. (2022). Characterization of chemical compositions by a GC–MS/MS approach and evaluation of antioxidant activities of essential oils from Cinnamomum reticulatum Hay, *Leptospermum petersonii* bailey, and Juniperus formosana Hayata. Arabian Journal of Chemistry.

[bb0115] Lin Y., Yu G., Zhang S., Zhu G., Yi F. (2024). Comparative analysis of the differences in volatile organic components of three lavender essential oils in Ili region using sensory evaluation, GC-IMS and GC-MS techniques. Journal of Chromatography A.

[bb0120] Liu D., Bai L., Feng X., Chen Y.P., Liu Y. (2020). Characterization of Jinhua ham aroma profiles in specific to aging time by gas chromatography-ion mobility spectrometry (GC-IMS). Meat Science.

[bb0125] Madhumita M., Guha P., Nag A. (2019). Extraction of betel leaves (*Piper betle* L.) essential oil and its bio-actives identification: Process optimization, GC-MS analysis and anti-microbial activity. Industrial Crops and Products.

[bb0130] Mdmc A., La B., La A. (2020). Usefulness of GC-IMS for rapid quantitative analysis without sample treatment: Focus on ethanol, one of the potential classification markers of olive oils - ScienceDirect. LWT.

[bb0135] Megumi C., Muroyama K., Sasako H., Tsuge N. (2017). Preventive activity of ar-Turmerone and Bisacurone isolated from turmeric extract against ethanol-induced hepatocyte injury. Food Science and Technology Research.

[bb0140] Nagarajan S., Kubra I.R., Rao L. (2010). Separation of Curcuminoids enriched fraction from spent turmeric oleoresin and its antioxidant potential. Journal of Food Science.

[bb0145] Nair K.P. (2013). The agronomy and economy of turmeric and ginger.

[bb0150] Pirbalouti A.G., Mahdad E., Craker L. (2013). Effects of drying methods on qualitative and quantitative properties of essential oil of two basil landraces. Food Chemistry.

[bb0155] Priyadarshi R., Ezati P., Rhim J.W. (2021). Recent advances in intelligent food packaging applications using natural food colorants. ACS Food Science & Technology.

[bb0160] Pushpa P., Gagana M.D., Santhosha K.M., Chandrarekha C., Yadava C.G. (2022). Economic assessment of black pepper under hilly zone multi-storyed ecosystem of Karnataka, India. International Journal of Environment and Climate Change.

[bb0165] Reddy Sagili S.U.K., Addo P.W., Gladu-Gallant F.-A., Bilodeau S.E., MacPherson S., Paris M., Orsat V. (2023). Optimization of wiped-film short path molecular distillation for recovery of cannabinoids from cannabis oil using response surface methodology. Industrial Crops and Products.

[bb0170] Scavarda C., Cordero C., Strocchi G., Bortolini C., Liberto E. (2020). Cocoa smoky off-flavour: A MS-based analytical decision maker for routine controls. Food Chemistry.

[bb0175] Sharma A., Bhardwaj G., Cannoo D.S. (2021). Antioxidant potential, GC/MS and headspace GC/MS analysis of essential oils isolated from the roots, stems and aerial parts of Nepeta leucophylla. Biocatalysis and Agricultural Biotechnology.

[bb0180] Shen D.Y., Li M.K., Song H.L., Zou T.T., Zhang L., Xiong J. (2021). Characterization of aroma in response surface optimized no-salt bovine bone protein extract by switchable GC/GC×GC-olfactometry-mass spectrometry, electronic nose, and sensory evaluation. LWT.

[bb0185] Shi Y., Chen G., Chen K., Chen X., Kan J. (2020). Assessment of fresh star anise (*Illicium verum* Hook.F.) drying methods for influencing drying characteristics, color, flavor, volatile oil and shikimic acid. Food Chemistry.

[bb0190] Soleimani V., Sahebkar A., Hosseinzadeh H. (2018). Turmeric (*Curcuma longa*) and its major constituent (curcumin) as nontoxic and safe substances: Review. Phytotherapy Research.

[bb0195] Wang R., Wen Q.H., Zeng X.A., Lin J.W., Li J., Xu F.Y. (2022). Binding affinity of curcumin to bovine serum albumin enhanced by pulsed electric field pretreatment. Food Chemistry.

[bb0200] Wang S., Chen H., Sun B. (2020). Recent progress in food flavor analysis using gas chromatography–ion mobility spectrometry (GC–IMS). Food Chemistry.

[bb0205] Xfab C., Hw A., Zwab C., Phab C., Jkab C. (2021). Discrimination and characterization of the volatile organic compounds in eight kinds of huajiao with geographical indication of China using electronic nose, HS-GC-IMS and HS-SPME-GC–MS. Food Chemistry.

[bb0210] Yao S., Wu S., Zhou Y., Liu Z., Tang M. (2021). Analysis of flavor components in HS-GC-IMS and antioxidant properties of black *Lycium barbarum* Rice wine. Journal of Food and Nutrition Research.

[bb0215] Yue G.L., Kwok H.F., Lee K.M., Jiang L., Chan K.M., Cheng L., Lau B.S. (2015). Novel anti-angiogenic effects of aromatic-turmerone, essential oil isolated from spice turmeric. Journal of Functional Foods.

[bb0220] Zhang L.L., Yang Z.Y., Fan G., Ren J.N., Yin K.J., Pan S.Y. (2019). Antidepressant-like effect of *Citrus sinensis* (L.) Osbeck essential oil and its main component limonene on mice. Journal of Agricultural and Food Chemistry.

[bb0225] Zhang S., Wu Y., Gao C., Wang Z., Li J., Li D. (2024). Process optimization and modeling of Acer truncatum seed oil deacidification by molecular distillation. LWT.

[bb0230] Zhao L., Yin L., Lei Z., Bu Y., Zhang C., Yang Y., Kong Y. (2017). Evaluation of quality changes of leisure dried tofu during storage based on electronic nose. Nanoscience and Nanotechnology Letters.

[bb0235] Zhou X., Chong Y., Ding Y., Gu S., Liu L. (2016). Determination of the effects of different washing processes on aroma characteristics in silver carp mince by MMSE–GC–MS, e-nose and sensory evaluation. Food Chemistry.

