# Supplementary material for: Comparative study on organoleptic properties and volatile organic compounds in turmeric, turmeric essential oil, and by-products using E-nose, HS-GC-IMS, and HS-GC-MS
Source: Food Chem X. 2024 Dec 28;25:102107. doi: 10.1016/j.fochx.2024.102107 (PMC11754684; doi:10.1016/j.fochx.2024.102107)
Supplement: Supplementary file 1 — Supplementary material (Table S1 and Figure S1) [file mmc1.docx]

Table S1 List of sensors and related compounds.

| **Sensors** | **Compounds** |
| --- | --- |
| Sensor 1 (W1C) | Aromatic compounds |
| Sensor 2 (W5S) | Nitrogen oxides |
| Sensor 3 (W3C) | Ammonia and aromatic compounds |
| Sensor 4 (W6C) | Hydrogen |
| Sensor 5 (W5C) | Alkanes and aromatic compounds |
| Sensor 6 (W1S) | Short-chain alkanes |
| Sensor 7 (W1W) | Sulfides and terpenes |
| Sensor 8 (W2S) | Alcohols, aldehydes, and ketones |
| Sensor 9 (W2W) | Organic sulfides and aromatic components |
| Sensor 10 (W3S) | Long-chain alkanes |

Fig. S1


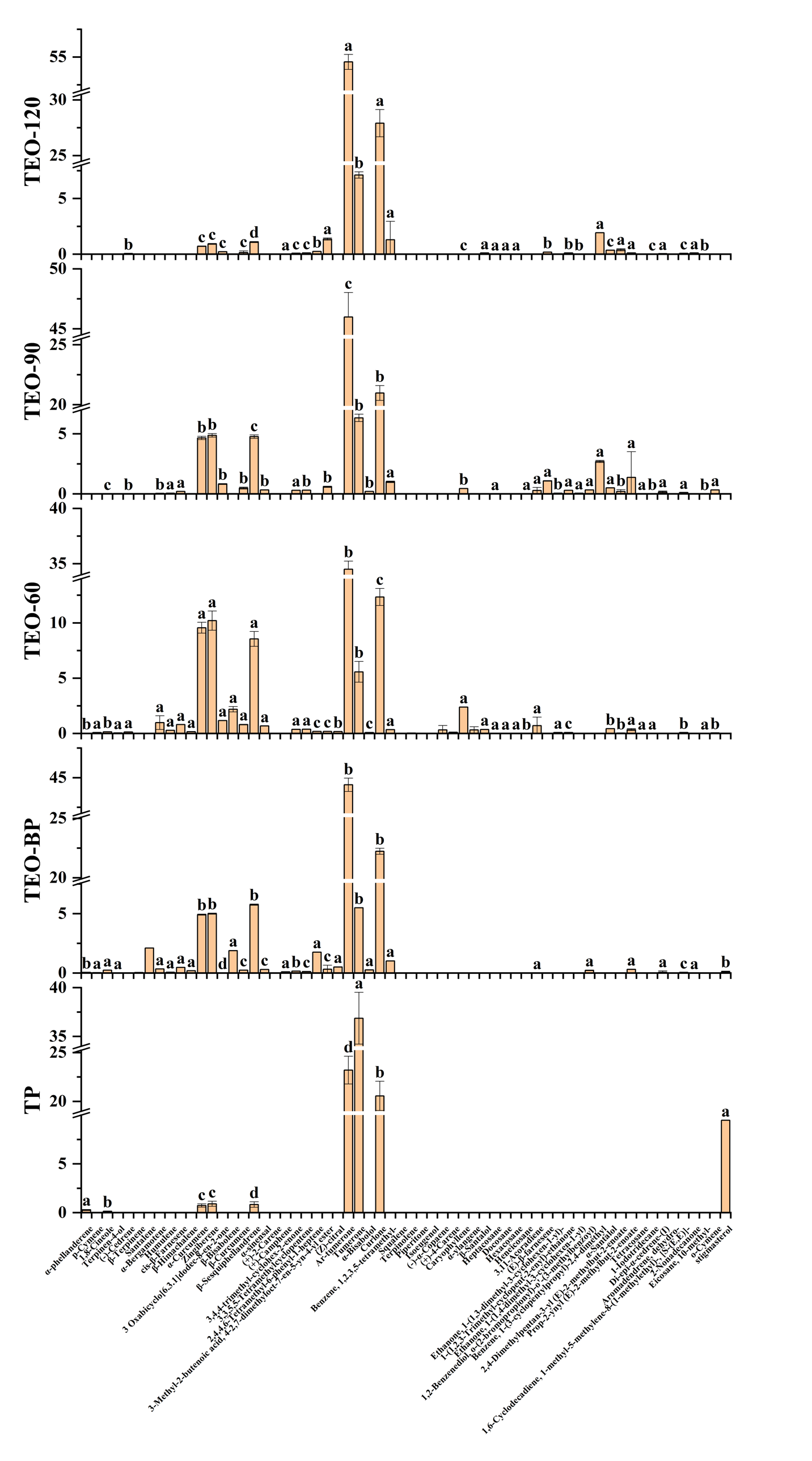


Fig. S1 Volatile organic compound composition and relative content of five different turmeric samples.
